# Drivers of exit and outcomes for Thoroughbred racehorses participating in the 2017–2018 Australian racing season

**DOI:** 10.1371/journal.pone.0257581

**Published:** 2021-09-21

**Authors:** Kshitiz Shrestha, James R. Gilkerson, Mark A. Stevenson, Meredith L. Flash

**Affiliations:** Faculty of Veterinary and Agricultural Sciences, Asia-Pacific Centre for Animal Health, The University of Melbourne, Parkville, Victoria, Australia; University of Illinois College of Veterinary Medicine, UNITED STATES

## Abstract

The destinations of Thoroughbred (TB) racehorses exiting the racing industry is a high-profile issue with ethical and welfare implications of interest to both animal welfare groups and racing regulators. This cross-sectional study investigated the reasons that TBs temporarily or permanently exited racing and training in Australia in the 2017–2018 racing season and the outcomes for these horses post-racing. An online questionnaire was sent to the last registered trainers of a representative sample of 2,509 ‘inactive’ TBs. Inactive horses were defined as those horses that were recorded as ‘active’ but had not trialled or raced in the last 6 months of the racing season or had an inactive status recorded in the Racing Australia database. Of the 1,750 responses received, the largest group of inactive TBs had permanently exited the racing industry (45% retired, 5.3% deceased). A relatively large group exited racing temporarily (43%) but participated in the racing industry in the following season. The reasons for retirement were predominantly voluntary, such as poor performance or owner’s request. Almost one third of retirements were due to injuries with tendon or ligament problems the most frequently conditions listed. The median age at retirement was five (Q1 4; Q3 7) years. Extrapolation of the survey results to the population of horses racing or training in Australia in 2017–2018 (*n* = 37,750) show that that 17% of the population retire each year and 2.1% die. These estimates provide benchmarks for industry and animal welfare organisations to resource and measure the effectiveness of interventions.

## Introduction

Globally, the use of animals in sport brings attention to the welfare standards under which these animals are managed. Community groups and industry stakeholders play an important role in influencing the compliance of animal sport industries with adequate welfare standards, by giving or withdrawing their acceptance or social license [1]. The Thoroughbred (TB) racing industry worldwide acknowledges that its continued social license is dependent on public perception of the standard of care of horses in the racing industry with the International Federation of Horseracing Authorities stating that a ‘horse welfare policy will only succeed after taking into account public perception’ [2]. Media coverage [3–6] of the fate of horses after leaving the TB industry highlights the extent to which the racing industry is scrutinised, although a recent population level study, investigating the outcomes for TB horses that left the racing industry, found that nearly two thirds retired to the TB breeding industry, or were re-homed outside of the TB industry [7].

The welfare of the horses in the care of the TB industry is of considerable interest to the general public, particularly issues related to the reasons that horses remain unraced, the racing of two-year-old horses and the outcomes for horses once they have retired from racing. In Australia, the most commonly reported barrier to entering racing was death which occurred most frequently before 1 year of age [8]. An Australian study conducted in 2014 investigated destinations and reason for horses leaving the racing stable [9] finding that 40% of horses left the stable in a year, however some of these exits were likely to be temporary such as spelling or transferred to another trainer. A representative, large-scale description of the outcomes of a representative sample of TB racehorses temporarily or permanently exiting racing stables in Australia is required. This will provide an evidence-base for the industry to engage in public debate around the welfare of the horses leaving the industry’s care, assist with resourcing programs aimed at transitioning horses to their post-racing career and evaluating the efficacy of such programs in the future.

The primary aim of this study was to identify the outcomes, reason and age of exit for horses that trained and raced in the 2017–2018 racing season in Australia. A secondary aim was to provide an estimate of the proportion of horses permanently leaving the Australian racing industry in a single racing season.

## Materials and methods

**Fig 1:**
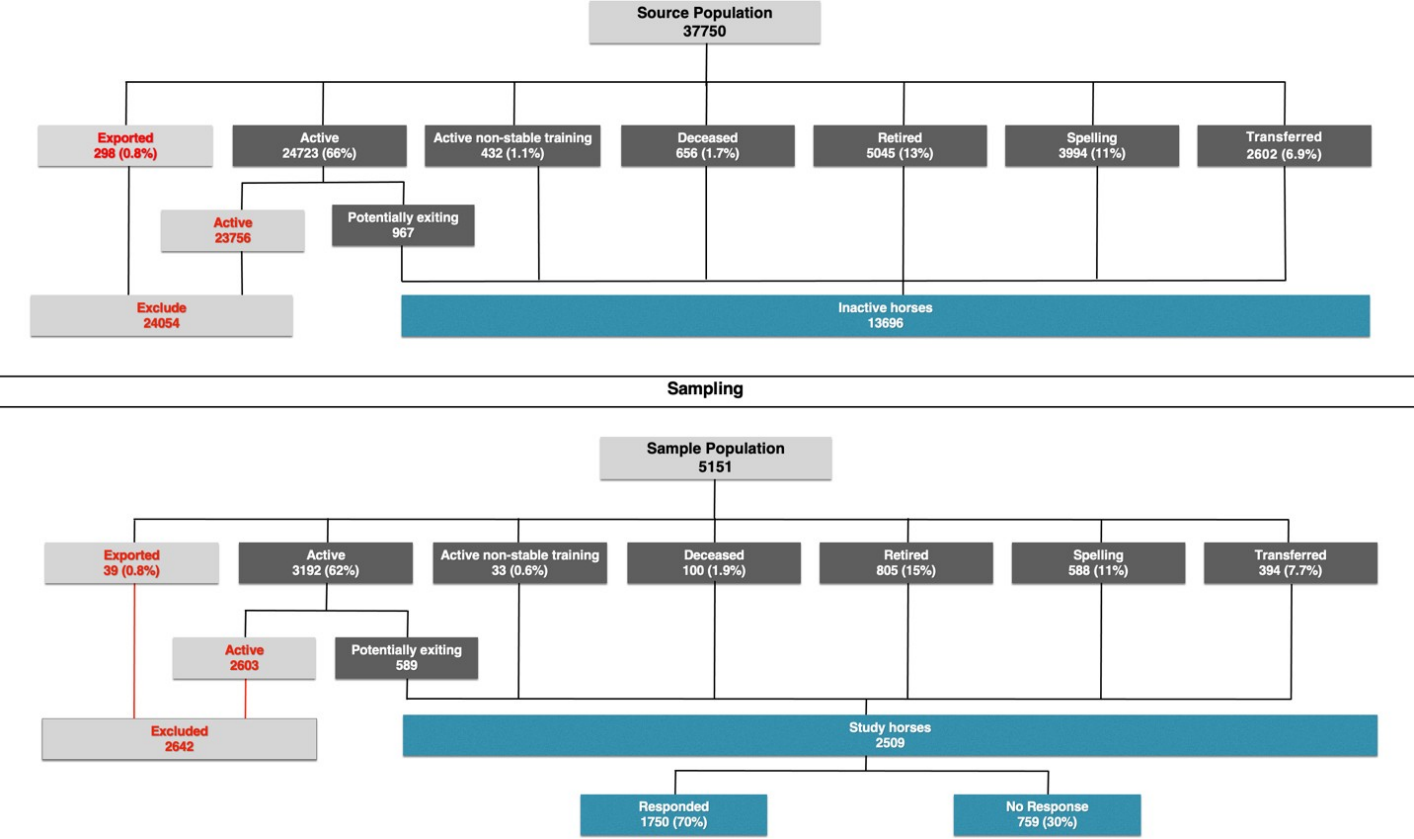
Flow chart of survey enrolment and response rate for the horses in 2017–2018 Australian Thoroughbred racing season.

This was a cross-sectional study of horses recorded by Racing Australia (RA) as racing and/or training in Australia in the 2017–2018 racing season to determine the proportions of horses alive and active within the TB racing industry (*P*_1_), the proportion of horses alive and active outside of the TB racing industry (*P*_2_), the proportion of horses that were exported (*P*_3_) and the proportion of horses that had died (*P*_4_). Having identified the horses that had left the TB racing industry in the 2017–2018 season (*P*_2_, *P*_3_ and *P*_4_) we sought to determine the reasons for their exit, classifying these into the following outcome groups: retired, deceased, participating in the racing industry and other (as listed in Fig 1).

The source population for this study comprised all TB horses recorded as racing and/or training in Australia in the 2017–2018 racing season by RA. Using the notation defined above we assumed that *P*_1_…*P*_4_ were mutually exclusive so that $\sum_{i=1}^{4}P_{i}=1$. To estimate the number of horses to enrol in this study we assumed that *P*_1_…*P*_4_ took the values 0.50, 0.40, 0.05 and 0.05 (respectively) and we wanted to sample a sufficient number of horses racing and/or training in Australia in the 2017–2018 racing season to be 95% certain that our estimates of the proportions of horses in each of the four outcome states were within 5% of the true (assumed) population values [10]. Based on these assumptions, a total of 3,268 horses were required to meet the specifications of the study. We further assumed that horse outcome states were not independent and were likely to be more similar within trainers and owners compared to between trainers and owners, so the crude sample size estimate of 3,268 was adjusted accordingly. An intra-class correlation coefficient *ρ* of 0.10 was conservatively assumed and it was also assumed that, on average, enquiries would be made concerning three horses in each training stable. This returned a design effect [11] of 1.2, so our crude sample size estimate was multiplied by this value to return an adjusted sample size of 3,268 × 1.2 = 3,922 horses. We anticipated that in the order of 30% of trainers would not respond to an invitation to take part in the study so the sample size of 3,922 was further increased to account for non-response returning a final sample size of 5,151 horses. Sample size calculations were completed using functions available in the contributed pps [12] and epiR [13] packages in R [14].

The postcode listed for the last stable return location was used to define the approximate location of each member of the source population. A stable return is a document that notifies RA of a horse’s activity while under the supervision of a licensed trainer. Generalized Random Tessellation Sampling (GRTS) [15] was used to select a spatially random sample of 5,151 horses from the source population which is referred to as the sampled population in the remainder of this paper. The enrolled population comprised horses with a reported inactive status (retired, spelling, deceased etc.), or were those that were recorded as active in the RA database, but had not trialled or raced in the second half of the racing season (these horses are referred to as potentially exiting, Fig 1). Surveys were administered to the last listed trainer identified from RA records of the enrolled population.

A total of 2,509 horses from the 5,151 sampled population were enrolled in the survey (the enrolled population, Fig 1). The study population comprised those members of the enrolled population for which a completed survey with a valid set of survey responses was received.

### Questionnaire

An online questionnaire was developed for the enrolled population using information obtained from ASB (Australian Stud Book) and RA records. A hyperlink to the questionnaire was sent via a personalised email to the last registered trainer for each member of the enrolled population on 24 December 2018. Follow-up phone calls commenced on 31 January 2019 to encourage a response from enrolled trainers who had not yet completed the survey. The survey closed on 24 June 2019.

The questionnaire asked trainers to nominate the horse’s current outcome status (still participating in the racing industry, re-homed/retired, deceased, other), age at the time of exit and reason for exit if the horse was no longer actively racing or training. Trainers were provided with options that were similar to those found on stable return lodgement forms, such as slow; illness or injury; owner request or proactive decision, and behaviour. They were further requested to specify the type of injury or illness, if injury or illness was selected as a reason for exit. The categories of injuries that trainers were asked to describe were broad, such as tendon or fracture. The options available for the trainers were those that were in common use to trainers and did not require in-depth veterinary input to accurately report these data.

Trainers had the option to select an ‘other’ category if the provided options were not deemed appropriate for questions relating to outcome, reason for exit and type of injury or illness leading to the outcome. These additional comments were reviewed and manually re-classified, where appropriate.

### Data analysis

**Fig 2:**
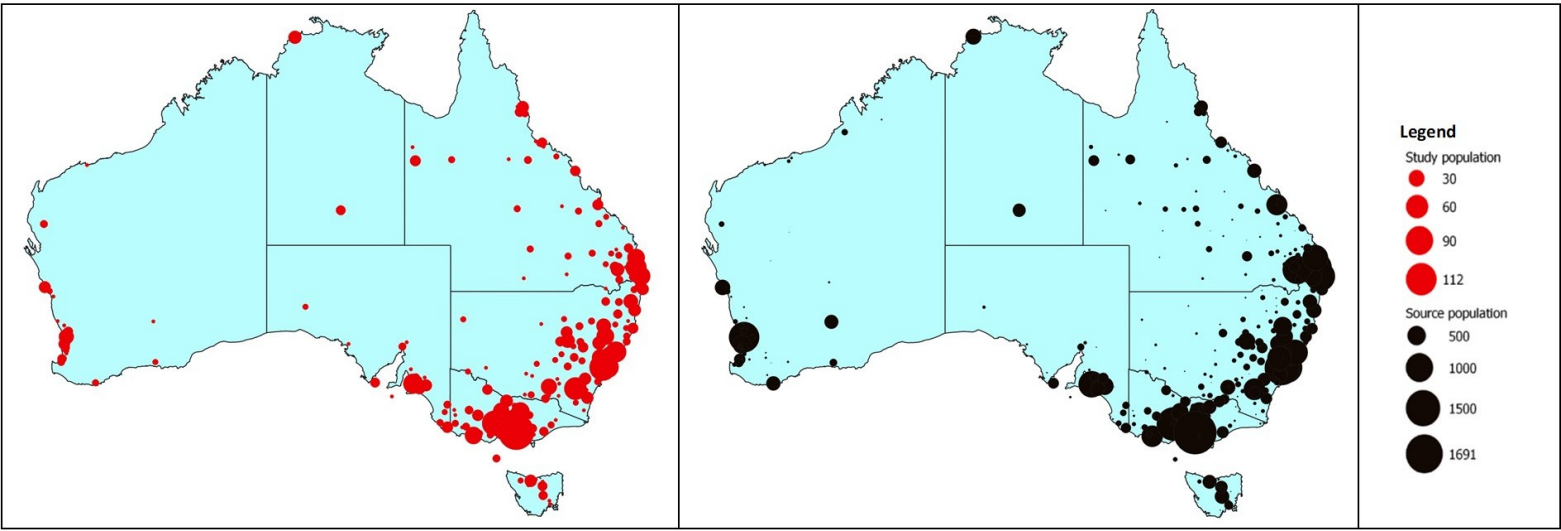
Map of Australia showing the postcode location of the study (●) population and the source (●) population for Australian Thoroughbred racing and training in the 2017–2018 racing season. Each point has been scaled by the number of horses at each location.

Comparisons of proportions were made using the chi-squared test. Horse ages were log transformed and horse ages in outcome groups were compared using the Student’s *t*-test. To determine whether horses in the study population were a representative sample of the source population the proportional similarity index (PSI) [16,17] and spatial scan statistic [18] were calculated. The PSI was used to quantify the agreement in the frequency distribution of source population horses stratified by state and by age with the frequency distribution of the study population stratified by state and by age. The spatial scan statistic was used to compare the geographic distribution of the study population with the geographic distribution of the source population. Similarity in the frequency distribution of source and study population horses stratified by state and by age provided reassurance that the study population was a truly random sample of the source population. Similarly, an absence of marked spatial clustering [19] of study population horses relative to the source population provided reassurance that the study population was a geographically representative sample of the source population (Fig 2).

The outcomes for this study are reported as counts and percentages for all survey responses and each outcome category, where appropriate.

For inferences made at the population level (i.e., estimates of the total number of horses that left the industry under the listed major exit categories across Australia) the data were analysed respecting the two-stage cluster sampling design used for this study. Due to more than 10% of trainers being sampled, a finite population correction factor for the first sampling stage (trainers) was applied. Trainer level and horse level sampling weights were calculated as the inverse probability of a trainer or horse being selected for study. Survey adjusted estimates of the proportion of horses participating in the racing industry (actively racing or training, ANST or spelling), rehomed or retired (either within or outside of the TB industry) or deceased (died, euthanised or sent to an abattoir) were calculated using the contributed survey package [20] in R. Survey adjusted proportions were then multiplied by the estimated number of inactive TBs across Australia in 2017–2018 (*n* = 13,696) to return estimates of the absolute number of horses in each category. These estimates were expressed as a fraction of the total number of TB horses registered with RA in 2017–2018 (*n* = 37,750).

The University of Melbourne Human Ethics Advisory Group approved this study and survey design (Application ID 1748714). All survey participants provided their informed consent to participate in the survey by completing the survey.

## Results

Of the 2,509 horses enrolled in the survey, responses were received for 1,750 (70%) horses from 933 trainers (Fig 1). The median number of horses per trainer in the sample population was one (Q1 1; Q3 3).

Of the 1,750 study horses, the majority (91%, 843 of 926) of the males were geldings. The median age of study horses was four (Q1 [quartile 1] 3; Q3 [quartile 3] 6) years. The PSI quantifying the similarity of the frequencies of the source population and the study population by state was 88% (CI 86% to 90%, S1 Table in S1 File). The PSI quantifying the similarity of the frequencies of the source population and the study population by age was 91% (CI 89% to 93%, S1 Table in S1 File).

Responses were not received for 759 horses from 321 trainers. Non-responses fitted into five categories: the trainer for 407 horses could not be reached and a message was left on their phone, trainers for 197 horses indicated they would respond later but had not completed the survey by the time it was closed, trainers for 78 could not be reached (due to disconnected or incorrect phone numbers, only their email address was recorded, or they were deceased or disqualified), and trainers for 41 horses had phone numbers with no option to leave a message. One percent (*n* = 36) of surveys were not completed because the participant chose to opt out.

Of the 1,750 study horses, 45% had permanently left the racing industry due to retirement or re-homing and 5% had died (Table 1). The majority of deceased horses in the study population died, or were euthanised, with only 3 horses reportedly sent to an abattoir (Table 1). Interestingly, 43% of study horses had only temporarily left the training stable and were participating in the racing industry in the following year (Table 1). The remaining 7% of study horses were categorised as exported, returned to owner, transferred or sold or the trainer was unable to provide an outcome (Table 1).

**Table 1:** Survey outcomes stratified by sex for 1,750 study horses classified as inactive in the 2017–2018 Australian Thoroughbred racing season.

Outcome	Female		Male		Total	
	*n*	(%)	*n*	(%)	*n*	(%)
Participating in the racing industry:						
Actively racing or training	274	(33)	387	(42)	661	(38)
ANST	2	(0.2)	8	(0.9)	10	(0.6)
Spelling	35	(4.2)	52	(5.6)	87	(5.0)
Subtotal	311	(38)	447	(48)	758	(43)
Rehomed/retired:						
Within the TB industry ^a^	233	(28)	9	(1)	242	(13)
Outside the TB industry	175	(21)	363	(39)	538	(31)
Subtotal	408	(49)	372	(40)	780	(45)
Deceased:						
Died or euthanised	31	(3.8)	62	(6.7)	93	(5.3)
Sent to abattoir	0	(0.0)	3	(0.3)	3	(0.2)
Subtotal	31	(3.8)	65	(7.0)	96	(5.3)
Other:						
Exported	19	(2.3)	13	(1.4)	32	(1.8)
Public or private sale	17	(2.0)	8	(0.9)	25	(1.4)
Returned to owner	14	(1.6)	9	(1.0)	23	(1.3)
Transferred	3	(0.4)	5	(0.5)	8	(0.5)
Unknown or unspecified	21	(2.5)	7	(0.8)	28	(1.6)
Subtotal	74	(9.0)	42	(4.5)	116	(6.6)
Total (%)	824	(47)	926	(53)	1750	(100)

^a^ Includes horses that retired as Australian Stud Book breeding stock (*n* = 234) and horses retired within the industry as clerk of the course horses or lead ponies (*n* = 8). ANST = Active Non-Stable Training; TB = Thoroughbred.

### Survey responses by age

**Fig 3:**
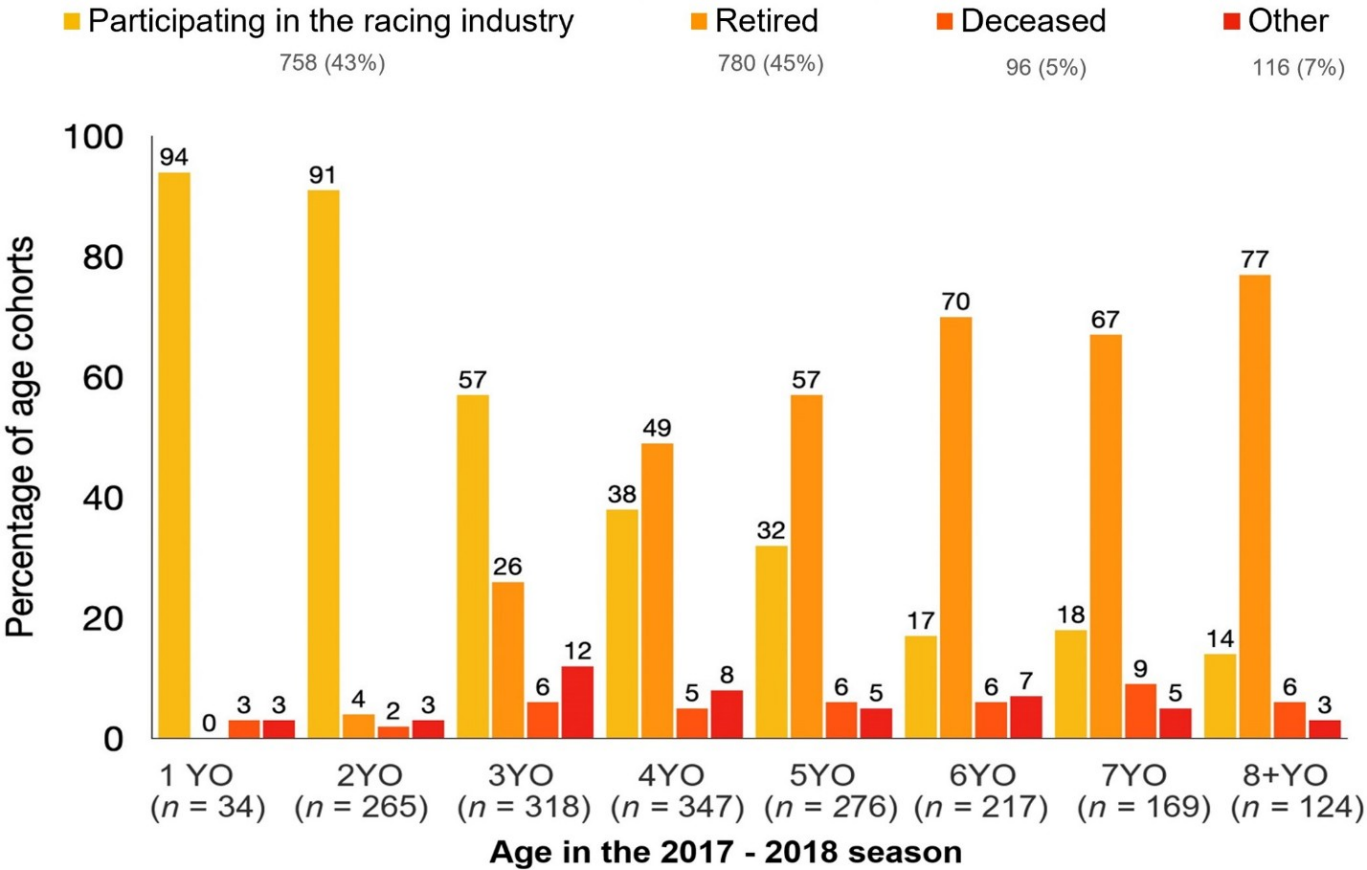
The percentage of survey outcomes by age for 1,750 study horses classified as inactive in the 2017–2018 Australian Thoroughbred racing season. Other: Exported, returned to owner, transferred, sold or unknown.

The largest age group in the study population were four-year-old horses, followed by those that were three-years, five-years, two-years and horses that were six-years of age and older (Fig 3). There were only 34 horses aged less than two years in the 2017–2018 racing season, the majority (94%) of these horses were participating in the racing industry (active, spelling or active non-stable training) the following season (Fig 3). The outcomes differed by age group, with horses aged one to four years more likely to be participating in the racing industry than horses that were five years of age or older (χ^2^ test statistic 266.918; *df* = 1; p <0.001) (Fig 3). Intact males were 2.1 times more likely to be aged one to four years than geldings (prevalence ratio [PR] 2.1; 95% CI 1.9 to 2.3; p <0.001). Conversely, horses aged five years and older were more likely to be identified as retired than other age groups (χ^2^ test statistic 259.690; *df* = 1; p <0.001) (Fig 3). Horses categorised as deceased accounted for between three and six percent of the outcomes across the age groups (Fig 3).

### Retirement

The median age of the 780 study horses categorised as retired was five (Q1 4; Q3 7) years. Females were 1.2 times more likely to be retired than males (PR 1.2; 95% CI 1.1 to 1.37; p <0.001). Geldings were 7.2 times more likely to be retired compared with intact males (PR 7.2; 95% CI 3.1 to 17; p <0.001). The median age of retirement at five (Q1 4; Q3 6) years for females was significantly less than median age of retirement for males at six (Q1 5; Q3 7) years (*t*-test statistic -8.22; *df* = 778; p < 0.001).

The most frequently reported activities undertaken by retired or re-homed horses were equestrian or pleasure horse activities (45%), followed by those that became ASB bloodstock and together these two categories accounted for three-quarters of all retirement outcomes (Table 2). Males were more likely to retire to undertake equestrian or pleasure pursuits than females (χ^2^ test statistic 211.34; *df* = 1; p <0.01, Table 2). Conversely, females were more likely to retire to ASB bloodstock activities than males (χ^2^ test statistic 293.963; *df* = 1; p <0.001, Table 2).

**Table 2:** Post-racing activities undertaken by survey horses reported as retired in the 2017–2018 Australian Thoroughbred racing season (*n* = 780).

Outcome	Female		Male		Total	
	*n*	(%)	*n*	(%)	*n*	(%)
Equestrian/pleasure	82	(20)	269	(72)	351	(45)
ASB Bloodstock	232	(57)	2	(0.5)	234	(30)
Companion	17	(4.1)	49	(13)	66	(8.4)
Broodmare for non-TB	36	(8.8)	0	(0)	36	(4.6)
Returned to owner	16	(3.9)	15	(4.0)	31	(3.9)
Industry	1	(0.2)	7	(1.8)	8	(1.0)
Other	3	(0.7)	9	(2.4)	12	(1.5)
Unknown or unspecified	22	(5.3)	24	(6.4)	46	(5.8)
Total	409 ^a^		375 ^b^		784 ^c^	

^a^ A total of 409 responses from 408 surveys: One survey categorised one mare as an equestrian and broodmare for non-TB

^b^ 375 responses from 372 surveys: Three surveys with multiple responses (equestrian and companion (*n* = 2), and companion and industry outcomes (*n* = 1)

^c^ 784 responses from 780 survey due to four survey having two retirement outcomes (equestrian and companion (*n* = 2), equestrian and broodmare for non-TB (*n* = 1) and companion and industry outcomes (*n* = 1); ASB = Australian Stud Book; TB = Thoroughbred.

#### Reasons for retirement

More than half (57%, 451 of 780) of the reasons given for retirement were for voluntary reasons, such as poor performance or owner request (Table 3). There was no statistically significant difference between the proportion of male and female horses that retired due to poor performance (χ^2^ test statistic 0.003; *df* 1; p = 0.96, Table 3). Male horses were more likely to retire due to injury or illness than females (χ^2^ test statistic 19.20; *df* 1; p <0.001, Table 3). There were 19 horses categorised as having been retired because they were ‘too old’, and these had a median age of eight (Q1 7, Q3 9) years.

**Table 3:** Reasons for retirement of the survey horses reported as retired, stratified by sex in the 2017–2018 Australian Thoroughbred racing season (*n* = 780).

Reason for retirement	Female		Male		Total	
	*n*	(%)	*n*	(%)	*n*	(%)
Poor performance	144	(35)	132	(35)	276	(35)
Injury/Illness	90	(22)	135	(36)	225	(29)
Owner request	111	(27)	64	(17)	175	(22)
Age	6	(1.5)	13	(3.5)	19	(2.4)
Behaviour	11	(2.7)	8	(2.2)	19	(2.4)
Lost interest in racing	4	(0.9)	7	(1.9)	11	(1.4)
Other	5	(1.2)	4	(1.1)	9^d^	(1.2)
Unknown or unspecified	41	(10)	15	(4.0)	56	(7.2)
Total	412 ^a^		378 ^b^		790 ^c^	

^a^ A total of 412 responses from 408 surveys: Four surveys with multiple responses (poor performance and behaviour (*n* = 1), injury/illness and behaviour (*n* = 1), injury/illness and lost interest in racing (*n* = 1), behaviour and other (*n* = 1)

^b^ 378 responses from 372 surveys: Six surveys with multiple responses (poor performance and behaviour (*n* = 1), owner request and injury/illness (*n* = 1), poor performance and injury/illness (*n* = 1), behaviour and lost interest in racing (*n* = 1), injury/illness and old (*n* = 1), poor performance and old (*n* = 1)

^c^ 790 responses from 780 surveys due to ten surveys having two reasons for retirement: (Poor performance and behaviour (*n* = 1), injury/illness and behaviour (*n* = 1), injury/illness and lost interest in racing (*n* = 1), behaviour and other (*n* = 1), (poor performance and behaviour (*n* = 1), owner request and injury/illness (*n* = 1), poor performance and injury/illness (*n* = 1), behaviour and lost interest in racing (*n* = 1), injury/illness and old (*n* = 1), poor performance and old (*n* = 1)

d. Other includes given responses: End of career (*n* = 3), broodmare value (*n* = 1), lease expired (*n* = 1), owner dispute (*n* = 1), owner illness (*n* = 1), trainer retirement (*n* = 1), small (*n* = 1).

Of the 29% (225 of 780) of retired horses where the reason for retirement was listed as injury or illness, musculoskeletal type injuries accounted for nearly three-quarters of all responses, with tendon or ligament injury (39%) being the most frequently reported injury (Table 4). The median age for horses retiring due to injuries or illnesses overall was five (Q1 4; Q3 6) years, however horses retired due to tendon/ligament injury had a median age of six (Q1 5; Q3 7) years. There was no significant difference in the proportion of males exiting the industry due to tendon or ligament injuries compared with females (χ^2^ test statistic 1.80; *df* 1; p = 0.18). Interestingly, nearly half (46%, 103 of 225) of the horses that retired due to injury subsequently undertook equestrian pursuits after their retirement from racing, while 24% (*n* = 55 of 225) went on to become ASB bloodstock. Musculoskeletal injuries or illnesses classified as ‘other’ included arthritis (*n* = 12), joint issue (*n* = 10), lameness (*n* = 8), hoof issues (*n* = 4), muscle issue (*n* = 3), back issues (*n* = 2), shin sore (*n* = 2) and miscellaneous (*n* = 9).

**Table 4:** Injuries or illnesses leading to retirement of the survey horses reported as retired, stratified by sex in the 2017–2018 Australian Thoroughbred racing season.

Illness or injury	Female		Male		Total	
	*n*	(%)	*n*	(%)	*n*	(%)
Musculoskeletal						
Tendon or ligament	30	(33)	57	(42)	87	(39)
Fracture	16	(18)	10	(7.4)	26	(12)
Other	26	(29)	24	(18)	50	(22)
Upper respiratory tract	2	(2.2)	20	(14)	22	(10)
Lower respiratory tract	4	(4.4)	13	(9.6)	17	(7.6)
Other	8	(8.9)	4	(3.0)	12	(5.3)
Unknown or unspecified	4	(4.4)	7	(5.2)	11	(4.9)
Total	90		135		225	

#### Equestrian activities for retired horses

Participation in pleasure horse activities post-racing was the most frequently reported equestrian activity, followed by show jumping, eventing, pony club, adult riding, dressage, show horse and polo (Table 5). There was no significant difference in the proportion of females compared with males involved as a pleasure horse (χ^2^ test statistic 0.57; *df* 1; p = 0.45).

**Table 5:** Equestrian activities undertaken by 351 study horses retired to equestrian or pleasure horse activities, stratified by sex in the 2017–2018 Australian Thoroughbred racing season.

Equestrian activity	Female		Male		Total	
	*n*	(%)	*n*	(%)	*n*	(%)
Pleasure horse	27	(33)	101	(38)	128	(36)
Show jumping	11	(13)	49	(18)	60	(17)
Eventing	8	(9.8)	39	(14)	47	(13)
Pony club	12	(15)	33	(12)	45	(13)
Adult riding	7	(8.5)	27	(10)	34	(9.7)
Dressage	11	(13)	22	(8.2)	33	(9.4)
Showhorse	6	(7.3)	12	(4.5)	18	(5.1)
Polo	10	(12)	7	(2.6)	17	(4.8)
Other	5	(6.1)	14	(5.2)	19	(5.4)
Unknown or unspecified	6	(7.3)	15	(5.6)	21	(5.9)
Total	103 ^a^		319 ^b^		422 ^c^	

^a^ 103 responses from 82 questionnaires due to 11 horses having multiple responses

^b^ 319 responses from 269 questionnaires due to 50 horses having multiple responses

^c^ 422 responses from 351 questionnaires due to 71 horses having multiple responses.

### Participating in the racing industry

The second most frequent outcome for the 1,750 study horses was that 43% of them were reported to be still participating in the racing industry (actively training or racing, spelling or undertaking active non-stable training). Males were more likely to be categorised as participating in the racing industry compared with females (χ^2^ test statistic 19.69; *df* 1; p <0.01, Table 1). Of the 758 horses participating in the racing industry, the majority (87%) were categorised as actively racing or training (Table 1). Overall, 5% (97 of 1,750) of survey responses identified horses as undertaking racing industry activities outside licensed training stables, such as spelling or active non-stable training (Table 1). The median age for these 758 horses participating in the racing industry was three (Q1 2; Q3 4) years.

### Deceased

Only 5% (*n* = 96) of the 1,750 survey responses categorised the study horse as deceased (Table 1). The median age of death was five (Q1 3; Q3 6) years. Males (68%, 65 of 96) were more likely to be listed as deceased compared with females (χ^2^ test statistic 8.92; *df* 1; p <0.01).

#### Circumstances and illness/injury leading to death

The majority of deceased horses (94%, 90 of 96) were categorised as dying due to injury or illness, with only one horse recorded as deceased due to behaviour. Of the three horses categorised as sent to an abattoir, all were geldings. The reason given for these geldings to be sent to abattoir were behaviour issues (*n* = 1, seven-year-old) and unspecified injury/illness during training (*n* = 2, aged four and seven-years). Injury incurred while exercising was the most frequently cited individual circumstance of death (54%, 49 of 90) collectively, with those dying during a race (*n* = 24) the most frequent, followed by deaths while training (*n* = 19) and while participating in a trial (*n* = 6, Table 6).

**Table 6:** Circumstances of death stratified by type of injury or illness for *n* = 93 survey horses that were reported as either died or euthanised, or sent to abattoir in the 2017–2018 Australian Thoroughbred racing season.

Injury/Illness	Deceased circumstances			
	Exercising	Non-exercising	Unspecified	Total
	*n*	*n*	*n*	(%)
Musculoskeletal:				
Fracture	27	10	0	37 (39)
Tendon or ligament	3	4	1	8 (8.4)
Other	0	4	0	4 (4.2)
Cardiac:	7	0	2	9 (9.5)
Digestive:	1	5	2	8 (8.4)
Infection:	2	1	0	3 (3.2)
Wounds or trauma:	1	2	0	3 (3.2)
Congenital malformation:	1	0	1	2 (2.1)
Respiratory:				
Upper	1	1	0	2 (2.1)
Lower	2	0	0	2 (2.1)
Other	3	4	2	9 (9.5)
Unknown or unspecified	0	2	6	8 (8.4)
Total	48	33	14	95 ^a^

^a^ Two horses had two injury/illness nominated.

Musculoskeletal injuries were the most frequently (54%, 49 of 90) cited injury or illness, with fractures, the most frequent individual injury resulting in death (Table 6). The majority of fractures (27 of 37) were categorised as occurring during exercise. Injuries and illnesses classified as ‘other’ included surgical complications (*n* = 2), cancer (*n* = 1), electrocuted (*n* = 1), foaling issues (*n* = 1), ocular cyst (*n* = 1), suspected snakebite (*n* = 1), immune (clotting disorder, *n* = 1), multiple-drug resistant infection (*n* = 1). Trainers were not able to provide the specified injury or illness leading to death for 5% (5 of 96) of deceased horses.

### Other outcomes

The median age of the 25 horses sold at a private or public sale was three (Q1 3; Q3 4) years. The most frequent reason for sale was poor performance (44%, *n* = 11), followed by injury or illness (*n* = 3), owner’s request (*n* = 2), age (*n* = 1) and behaviour (*n* = 1) with the remaining seven responses unknown or unspecified.

There was no statistically significant difference between male and female horses exported (χ^2^ test statistic 0.37; *df* 1; p = 0.54). Similarly, no statistically significant difference between sexes was observed for horses with unknown outcomes (χ^2^ test statistic 2.00; *df* 1; p = 0.16) or sold (χ^2^ test statistic 0.24; *df* 1; p = 0.62).

### Potentially exiting horses

The enrolled population (*n* = 2,509) included 589 horses, with an ‘active’ status with RA at the end of the 2017–2018 racing season, that had not trialled or racing in the last six months of the season (Fig 1, potentially exiting). Of these 589 potentially exiting horses, more than half (56%, *n* = 332 of 589) were males. The median age of potentially exiting horses was four (Q1 3; Q3 6) years, which was similar to the larger study population. The response rate for the potentially exiting horses was slightly higher (79%, 463 of 589) than the response rate for the study overall.

Of 463 responses received for potentially exiting horses, the majority (60%, *n* = 278) were categorised as still participating in the racing industry, followed by 33% (*n* = 152) retired, 3% (*n* = 13) deceased, 1% (*n* = 6) exported, 1% (*n* = 5) returned to owner, 1% (*n* = 2) sold and 1% (*n* = 2) unknown. The median age of these horses that were categorised as participating in the racing industry was three (Q1 2; Q3 5) years, and retired horses was five (Q1 4; Q3 7) years, similar to the rest of the survey responses.

### Extrapolation of survey results to the 2017–2018 source population

Analyses respecting the two-stage cluster sampling design used for this study (with individual horses clustered within trainers) allowed survey outcomes to be extrapolated to horses from the 2017–2018 source population that met the ‘inactive’ horses (*n* = 13,696, Fig 1), providing estimates of the absolute number of horses in each of the major outcome groups. Extrapolation of the survey outcomes showed that 47% (6,380 of 13,696) of inactive TBs in the 2017–2018 season in Australia were rehomed or retired and a further 5.8% (794 of 13,696) died or were euthanised (Table 7). The estimate that 41% (5,624 of 13,696) inactive TBs had only temporarily exited the racing stable and were still participating in the racing industry, by either actively racing or training, spelling or undertaking active non-stable training the following season is important for both regulators and the public alike.

**Table 7:** Extrapolation of survey results to 13,696 Thoroughbred racehorses classified as inactive from the source population in the 2017–2018 Australian racing season.

Outcome	Survey (*n*)	% (95% CI)	Estimated counts (95% CI) ^a^
Participating in the racing industry:			
Actively racing or training	661	36 (33, 38)	4903 (4555, 5259)
ANST	10	0.6 (0.3, 1.0)	81 (42, 139)
Spelling	87	4.7 (3.7, 6.0)	639 (512, 785)
Subtotal	758	41 (38, 44)	5624 (5256, 5996)
Rehomed or retired:			
Within the TB industry	234	13 (12, 15)	1845 (1624, 2083)
Outside the TB industry	546	33 (31, 36)	4535 (4186, 4893)
Subtotal	780	47 (44, 49)	6380 (6003, 6758)
Deceased:			
Died or euthanised	93	5.6 (4.6, 7.0)	766 (625, 926)
Sent to abattoir	3	0.2 (0.06, 1.0)	28 (8, 67)
Subtotal	96	5.8 (4.7, 7.0)	794 (651, 956)
Other:			
Exported	32	1.7 (1.1, 2.0)	231 (154, 330)
Public or private sale	25	1.2 (0.1, 2.0)	172 (112, 249)
Returned to owner	23	1.3 (0.8, 2.0)	178 (114, 262)
Transferred	8	0.5 (0.2, 1.0)	70 (31, 132)
Unknown or unspecified	28	1.8 (1.1, 3.0)	248 (156, 370)
Subtotal	116	6.6 (5.4, 8.0)	899 (738, 1079)

^a^ Based on the number of TB horses estimated to be inactive in Australia in the 2017–2018 racing season (*n* = 13,696).

## Discussion

Racing Australia data showed that in the 2017–2018 season 37,750 TB horses raced and trained. Based on the results of the cross-sectional study presented in this paper, we estimate that 17% of these horses permanently left the racing industry through retirement and a further 2.1% died or were euthanised in this season. The temporary nature of some stable exits was highlighted by our finding that nearly 6,000 of horses classified by RA as inactive were actually still participating in the racing industry, by either racing or training, spelling or undertaking active non-stable training the following season. Horses exiting a training stable temporarily for spelling or active non-stable training reasons are normal and necessary pauses in a TBs racehorse’s training schedule to allow rest and recuperation from the rigours of race training. Previous Australian research that investigated the 2010 Victorian foal crop found that nearly three quarters of the foal crop were retired by eight years of age [7]. The estimate that 19% of the horses training and racing in Australia permanently leave the racing industry each year either through retirement (17%) or death (2.1%) is lower than previous Australian research [9,21]. This may be due to the inclusion of temporary exits in the previous estimates. Racehorses exiting a stable for spelling or active non-stable training activities are part of the normal training management of TB racehorses.

The 47% of horses categorised as rehomed or retired in this study (Table 7) is similar to the 42% TBs reported by Thomson *et al* [9]. The median age of retirement in this study was five-years which agreed with the exit survey data for the 2010 Victorian foal crop [7]. Investigation of potentially exiting horses found a similar age of retirement, with a third of these horses retiring. This finding shows that regulators should focus their traceability efforts on TBs over five-years of age that had not raced or trialled in the last six months. While the age of retirement for females agrees with an earlier Australian study investigating the 2010 Victorian foal crop, males in this study were slightly older at the time of retirement [7].

Previous Australian and New Zealand studies [7,22] reported that the majority of retirements were voluntary in nature, often due to poor performance or owner requests, which is similar to the findings reported in this study. This relatively consistent age of retirement together with the majority of horses retiring for voluntary reasons suggests the decision to retire is not entirely depended on biological or physiological effects, rather it is due to a conflation of factors including horse performance and other industry-level effects. Similar to the Australian foal crop study [7], for those horses that retired due to injury or illness, nearly three quarters were due to musculoskeletal injury with tendon or ligament injuries the most frequent. These findings agree with a US study, that reported off-the-track TB horses (OTTB) were more likely to have disorders related to musculoskeletal and gastrointestinal, than non-OTTB controls [23]. It is important to note that while injury and illness were significant reasons for retirement from racing in the current survey, a little under one half of these ‘injured’ horses were undertaking ridden activities such as equestrian pursuits in retirement which was similar to the findings of previous Australian research [7]. Further studies on investigating the post-racing career length and health disorders that reduces the transition between careers are necessary.

There was a considerable difference in the post-racing activities of retired racehorses depending upon their sex. Females were more likely to be retired within TB racing industry as ASB bloodstock, while males were more likely to be involved in pleasure riding or equestrian activities. Previous Australian research has identified differences in career length between intact males and geldings [24]. Intact males in this study were 2.1 times more likely to be aged four years or younger and geldings were 7.2 times more likely to be retired than intact males. With only two males retiring to Australian Stud Book duties, our findings suggest that the career length differences are likely due to males being gelded once they are identified as not being stallion prospects.

The design of this study ensured that those horses that were the subject of our investigations (the study population) were representative of the source population, as confirmed by our proportional similarity index and spatial scan statistic analyses. Representative sampling, in combination with analyses that respected the hierarchical nature of the data (that is, individual horses clustered within trainers) allowed us to extrapolate the findings from this study back to the source population in both relative and absolute terms. We estimate that in the order of 4,500 horses retired outside of the jurisdiction of the TB racing and breeding industries in the 2017–2018 racing season (Table 7). The lack of jurisdictional oversight of these horses by racing authorities presents a number of challenges. Currently, the racing industry is not able to trace these horses once they leave the care of a licensed participant. The finding that horses are retiring at a relatively young age means that these horses spend a large part of their lives outside of the industry’s oversight, while still presenting a risk to the industry’s social license. For horses that were sold, transferred or were returned to their owners, the trainer was unable to provide any further detail on the horse’s location or activities, which also represents a gap in traceability.

The proportion of horses that met the criteria for inclusion in this study that were deceased (5.8%, 95% CI 4.7% to 7.0%, Table 7) is consistent with a previous Australian study carried out in 2014 [9]. The proportion of horses deceased was relatively consistent across all age groups. The survey findings indicated that approximately half of the fatalities occurred during training or racing activities with fracture most frequent injury reported. These are consistent with the hypothesis that intense exercise without appropriate bone re-modelling may predispose horses to catastrophic injury [25–27]. In agreement with previous studies, males were more likely to die than females due to fractures [27].

An unexpected finding was the relatively small number of horses that were sent to an abattoir (0.2%, 95% CI 0.06% to 1.0%, Table 7). This was lower than that reported by Racing Australia [28] and may reflect changes in the rules of racing in 2016 that aim to prevent horses going to knackeries and abattoirs directly from racing.

Previous studies in Australia examined the health condition that interfered with training and identified that respiratory disease and shin soreness were the most frequently reported concerns to Australian trainers, as it resulted interruption to the training schedule for individual horses [29]. Interruptions due to these conditions were likely to be temporary. Because this study documented the reasons why horses leave the racing industry with a focus on a horse’s permanent exit, it is not surprising that more serious health conditions such as tendon or ligament injuries and fractures were reported more frequently compared to previous research.

The limitations of this study were restricted to those of all surveys, that responses provided by survey participants are liable to recall bias and rely on the memory or good record keeping. The 933 trainers undertaking the survey for 1,750 horses and the relatively short follow-up period would have helped trainers to recall details of specific horses and reduced the influence of recall bias for this study. The potential for selection bias was minimised in this study through the use of the GRTS sampling method which meant that a spatially representative sample was collected from the TB horse population across Australia.

## Conclusions

This study provides a cross-sectional estimate of the outcomes for inactive horses in the 2017–2018 racing season and provides population level estimates of horses temporarily and permanently exiting the racing stable. The permanent outcomes of retired or deceased for these TBs was lower than previous Australian research. This is likely due to the inclusion of temporary stable exits in the estimates from previous research. The median age of horses ‘voluntarily’ retiring at five years is contrary to the belief that horses are forced to retire mainly due to injury. The retirement of horses at a relatively young age with the majority moving outside the industry’s jurisdiction is an ongoing risk to TB racing’s social license. The extrapolation of these data to the broader racing population in 2017–2018 provide the racing industry with a benchmark to assist with resourcing and evaluating programs aimed at incentivising traceability of TBs moving outside its jurisdiction into the wider horse industry.

## Supporting information

S1 File Analysis of source population and S1 Table. Source Thoroughbred horse population as a function of age in the 2017–2018 Australian Thoroughbred racing season, stratified by state or place of origin and age group.
(PDF)

S2 File Australian Thoroughbred Wellbeing Survey and link to an example survey. (PDF)

S3 File Dataset. (CSV)
