# Supplementary material for: Drivers of exit and outcomes for Thoroughbred racehorses participating in the 2017–2018 Australian racing season
Source: PLoS One. 2021 Sep 21;16(9):e0257581. doi: 10.1371/journal.pone.0257581 (PMC8454983; doi:10.1371/journal.pone.0257581)
Supplement: S1 File — Source Thoroughbred horse population as a function of age in the 2017–2018 Australian Thoroughbred racing season, stratified by state or place of origin and age group. (PDF) [file pone.0257581.s001.pdf]

Supplementary Item 1

Analysis of the source population age

The source population for this study comprised a total of 37,750 TB horses racing and/or training recorded by RA in Australia in the 2017-2018 racing season. Of these, 101 horses were imported to Australia during the 2017-2018 racing season. The age of these horses ranged from one to twelve-years. The median age of the source population was four years (Q1 [quartile 1] 3; Q3 [quartile 3] 5) years. Horses three years of age comprised the largest proportion (23%) of the source population, followed by four-years of age, with two-years and five years an equal 16% of the population (S1 Table 1). Horses aged six-years and older comprised over one-fifth of the population (21%, S1 Table 1). While Victoria had the second largest proportion of horses over all in training or racing, it had the largest proportion of two-year-old (36%, 2,172 of 6,080) and horses eight-years of age and older (29%, 566 of 1,935).

Supplementary Table 1. Source Thoroughbred horse population as a function of age in the 2017- 2018 Australian Thoroughbred racing season, stratified by state or place of origin and age group.

State	1 YO	2 YO	3 YO	4 YO	5 YO	6 YO	7 YO	8+YO	Total n (%)
NSW	22	1,929	2,802	2,590	1,815	1,142	609	463	11,372 (30)
VIC	331	2,172	2,652	2,138	1,428	887	548	566	10,722 (28)
QLD	0	933	1,523	1,692	1,352	921	543	407	7,371 (20)
WA	0	630	961	929	702	476	264	238	4,200 (11)
SA	2	292	531	520	428	283	198	191	2,445 (6)
TAS	0	110	236	241	187	141	81	33	1,029 (3)
NT	0	13	87	121	120	86	56	27	510 (1)
Imported	0	1	23	17	27	14	9	10	101 (0.2)
Total n (%)	355 (1)	6,080 (16)	8,815 (23)	8,248 (22)	6,059 (16)	3,950 (10)	2,308 (6)	1,935 (5)	37,750 (100)

Abbreviations: NSW: New South Wales, VIC: Victoria, QLD: Queensland, WA: Western Australia, SA: South Australia, TAS: Tasmania, NT: Northern Territory; YO = Years old
