# Supplementary material for: Drivers of exit and outcomes for Thoroughbred racehorses participating in the 2017–2018 Australian racing season
Source: PLoS One. 2021 Sep 21;16(9):e0257581. doi: 10.1371/journal.pone.0257581 (PMC8454983; doi:10.1371/journal.pone.0257581)
Supplement: S2 File — (PDF) [file pone.0257581.s002.pdf]

Supplementary Item 2

Hyperlink to online example survey:

https://www.surveymonkey.com/r/TK2MS6Q?&n=Fast%20As&s=Quick%20Boots&d=Lightning%20McQueen&sx=Male&db=1/5/2016&cr=Pink%20Striped

Australian Thoroughbred Wellbeing Survey

Horse information

Records obtained from the Australian Stud Book and Racing Australia identify you as the registered breeder, owner, or trainer of:

Life number: (prefilled data)

Name: (prefilled data)

Sire: (prefilled data)

Dam: (prefilled data)

Sex: (prefilled data)

Colour: (prefilled data)

Date of Birth: (prefilled data)

To the best of your knowledge, is “horsename” (prefilled data) currently:

(A) ☐ Actively racing/Race training (2018/2019 season)
(B) ☐ Active non-stable training (i.e. pretraining, water walker, etc.) (2018/2019 season)
(C) ☐ Spelling
(D) ☐ Australian stud book mare/stallion
(E) ☐ Rehomed/Retired
(F) ☐ Livestock sale/private sale
(G) ☐ Exported
(H) ☐ Deceased
(I) ☐ Other - please state in the box below

DECISION TREE AND QUESTIONS

Options (A) and (I)
Actively racing/Race training, Other

1. FINAL COMMENTS (See page 5)

Options (B) and (C)
Active non-stable training
Spelling

1. What date did the horse begin its spelling/non-stable training period?
2. What is the intended return to race training date if known?
3. If spelling, what is the reason for spelling?
   - Injury/Illness
   - Other (please specify)

3a. (If "injury/illness" was selected)
What was the injury or illness? Once you have selected an option please comment on the specific injury, if known, as well as any additional information you might have in the comments box below.
- Fracture
- Tendon/Ligament injury
- Upper respiratory condition i.e. roarer, displaced soft palate, strangles, etc.
- Lower respiratory condition i.e. bleeder, asthma, pneumonia, etc.
- Cardiac/metabolic condition i.e. arrhythmia, haemorrhage, etc.
- Digestive condition i.e. colic, peritonitis, etc.
- Congenital malformation i.e. contracted tendons, wobbler, etc.
- Immune condition i.e. neonatal isoerythrolysis, etc.
- Other (please specify)

4. FINAL COMMENTS (See page 5)

Options (D) and (F)
Australian Stud Book mare/stallion
Livestock sale/private sale

1. If horse was not raced or trialed prior to the chosen outcome, had the horse undergone any training/pretraining?
   - Yes
   - No
   - N/A
2. At what age did the horse leave the industry?
3. Why did the horse leave the industry? Once you have selected an option, please provide us with any additional information in the comments box below. If you select "injury/illness" please proceed to the next page for more follow-up questions
   - Injury/Illness
   - Poor performance/slow
   - Unsuitable temperament/behaviour
   - Owner request/proactive decision
   - Other – please specify

3a. (If "injury/illness" was selected)
What was the injury or illness? Once you have selected an option please comment on the specific injury, if known, as well as any additional information you might have in the comments box below.
- Fracture
- Tendon/Ligament injury
- Upper respiratory condition i.e. roarer, displaced soft palate, strangles etc.
- Lower respiratory condition i.e. bleeder, asthma, pneumonia, etc.
- Cardiac/metabolic condition i.e. arrhythmia, haemorrhage, etc.
- Digestive condition i.e. colic, peritonitis, etc.
- Congenital malformation i.e. contracted tendons, wobbler, etc.
- Immune condition i.e. neonatal isoerythrolysis, etc.
- Other (please specify)

4. FINAL COMMENTS (See page 5)

Option (G)
Exported

1. At what age was the horse exported?
2. What country was the horse exported to?
3. FINAL COMMENTS (See page 5)

Option (E)
Rehomed/Retired

1. Please check the box most applicable to the outcome of the horse. Please provide us with specifics/any additional information you might have in the additional comments box below.
   ☐ Equestrian and pleasure pursuits: Dressage, show jumping, eventing, pony club, adult riding, trail riding, etc. If you have selected this option, please proceed to the next page for more follow-up questions.
   ☐ Companion or other un-ridden activities – please state what these are in the box below
   ☐ Broodmare for non-thoroughbreds
   ☐ Re-homed within the racing industry as a lead pony or clerk of the course
   ☐ Other – please state what these are in the box below
2. (If Equestrian and pleasure pursuits was selected)
   Which options best describe the equestrian/pleasure pursuits applicable to the horse? Please use the additional comments box to provide any additional details you might have about the individual pursuits.
   ☐ Dressage
   ☐ Show jumping
   ☐ Eventing
   ☐ Pony club
   ☐ Adult riding
   ☐ Pleasure horse/Hack
   ☐ Other – please state in the box below
3. If horse was not raced or trialed prior to the chosen outcome, had the horse undergone any training/pretraining?
   ☐ Yes
   ☐ No
   ☐ N/A
4. At what age did the horse leave the industry?
5. Why did the horse leave the industry? Once you have selected an option, please provide us with any additional information in the comments box below. If you select "injury/illness" please proceed to the next page for more follow-up questions
   ☐ Injury/Illness
   ☐ Poor performance/slow
   ☐ Unsuitable temperament/behaviour
   ☐ Owner request/proactive decision
   ☐ Other – please specify

5a. (If "injury/illness" was selected)
What was the injury or illness? Once you have selected an option please comment on the specific injury, if known, as well as any additional information you might have in the comments box below.
- ☐ Fracture
- ☐ Tendon/Ligament injury
- ☐ Upper respiratory condition i.e. roarer, displaced soft palate, strangles etc.
- ☐ Lower respiratory condition i.e. bleeder, asthma, pneumonia, etc.
- ☐ Cardiac/metabolic condition i.e. arrhythmia, haemorrhage, etc.
- ☐ Digestive condition i.e. colic, peritonitis, etc.
- ☐ Congenital malformation i.e. contracted tendons, wobbler, etc.
- ☐ Immune condition i.e. neonatal isoerythrolysis, etc.
- ☐ Other (please specify)

6. FINAL COMMENTS (See page 5)

Option (H)
Deceased

1. Which of the following best describes when the death of this horse occurred? Once you have selected an option, please provide us with any additional information you might have in the comments box below.
   ☐ Race
   ☐ Trial/jumpout
   ☐ Training/pretraining
   ☐ Other (please specify)
2. What was the horse's age of death?
3. What was the reason for death?
   ☐ Injury/Illness
   ☐ Sent to abattoir
   ☐ Other (please specify)

3a. (If "injury/illness" was selected)
What was the injury or illness? Once you have selected an option please comment on the specific injury, if known, as well as any additional information you might have in the comments box below.
- ☐ Fracture
- ☐ Tendon/Ligament injury
- ☐ Upper respiratory condition i.e. roarer, displaced soft palate, strangles etc.
- ☐ Lower respiratory condition i.e. bleeder, asthma, pneumonia, etc.
- ☐ Cardiac/metabolic condition i.e. arrhythmia, haemorrhage, etc.
- ☐ Digestive condition i.e. colic, peritonitis, etc.
- ☐ Congenital malformation i.e. contracted tendons, wobbler, etc.
- ☐ Immune condition i.e. neonatal isoerythrolysis, etc.
- ☐ Other (please specify)

OR

3b. (If "sent to abattoir" was selected)
What was the reason for sending to abattoir? Please provide us with further details, if known, in the comments box below.
- ☐ Owners request
- ☐ Behaviour
- ☐ Injury/Illness
- ☐ Performance
- ☐ Other (please specify)

4. FINAL COMMENTS (See page 5)

FINAL COMMENTS

Do you have any further comments regarding the outcome/current state of the horse?

Would you be happy for us to contact you if we have any further questions?

☐ **Yes**
☐ **No**

******** END OF SURVEY ********
